# A type 1 diabetes prediction model has utility across multiple screening settings with recalibration

**DOI:** 10.21203/rs.3.rs-5773430/v1

**Published:** 2025-02-06

**Authors:** Erin L. Templeman, Lauric A. Ferrat, Hemang M. Parikh, Lu You, Taylor M. Triolo, Andrea K. Steck, William A. Hagopian, Kendra Vehik, Suna Onengut-Gumuscu, Peter A. Gottlieb, Stephen S. Rich, Jeffery P. Krischer, Maria J. Redondo, Richard A. Oram, the Type 1 diabetes TrialNet Study Group

**Affiliations:** 1 Department of Clinical and Biomedical Sciences, University of Exeter, Exeter, UK; 2 Department of Genetic Medicine and Development, Univerisity of Geneva, Switzerland; 3 Health Informatics Institute, Morsani College of Medicine, University of South Florida, Tampa, FL, USA; 4 Barbara Davis Center for Diabetes, University of Colorado Anschutz Medical Campus, Aurora, CO, USA; 5 Pacific Northwest Research Institute, Seattle, WA, USA; 6 Department of Genome Sciences, University of Virginia School of Medicine, Charlottesville, VA, USA; 7 Baylor College of Medicine, Texas Children’s Hospital, Houston, TX, USA

**Keywords:** type 1 diabetes, prediction, recalibration, adjustment, genetics, autoantibody, FDR

## Abstract

**Background:**

Accurate type 1 diabetes prediction is important to facilitate screening for pre-clinical type 1 diabetes to enable potential early disease-modifying interventions and to reduce the risk of severe presentation with diabetic ketoacidosis. We aimed to assess the generalisability of a prediction model developed in children followed from birth. Additionally, we sought to create an application for easy calculation and visualization of individualized risk prediction.

**Methods:**

We developed and refined a stratified prediction model combining a genetic risk score, age, islet autoantibodies, and family history using data from children followed since birth by The Environmental Determinants of Diabetes in the Young (TEDDY) study. We tested the validity of the model through external validation in the Type 1 Diabetes TrialNet Pathway to Prevention study, which conducts cross-sectional screening in relatives of people with type 1 diabetes. We recalibrated the model by adjusting for baseline risk and selection criteria in TrialNet using logistic recalibration to improve calibration across all ages.

**Results:**

The study included 7,798 TEDDY and 4,068 TrialNet participants, with 305 (4%) and 1,373 (34%) developing type 1 diabetes, respectively. The combined model showed similar discriminative ability in autoantibody-positive individuals across TEDDY and TrialNet (p=0.14), but inferior calibration in TrialNet (Brier score 0.40 [0.38,0.43]). Adjustment for baseline risk and selection criteria in TrialNet using logistic recalibration improved calibration across all ages (Brier score 0.16 [0.14,0.17]; p<0.001). A web calculator was developed to visualise individual risk estimates (https://t1dpredictor.diabetesgenes.org).

**Conclusions:**

A stratified model of type 1 diabetes genetic risk score, family history, age, and autoantibody status accurately predicts type 1 diabetes risk, but may need recalibration according to screening stategy.

## Twitter Summary

🔬Exciting Diabetes Research Alert! 🔍We’re translating prediction estimates, paving the way for early intervention 🌟 👀 Stay tuned for an innovative application for personalized T1D risk calculation! 🚀#DiabetesResearch #HealthInnovation #T1DPrediction

Teplizumab, which is approved by the U.S. Food and Drug Administration (FDA) for stage 2 pre-clinical type 1 diabetes, has been shown to delay the onset of stage 3 (clinical) disease by a median of 3 years(1,2). The need to identify individuals eligible for therapy, coupled with growing evidence for lower risk of diabetic ketoacidosis in individuals identified in early stages of pre-clinical type 1 diabetes(3), has driven heightened interest in screening and risk prediction for type 1 diabetes. The stages of pre-clinical type 1 diabetes include individuals with multiple islet autoantibodies (stage 1), and individuals with evidence of dysglycaemia in addition to islet autoantibodies (stage 2)(4). A combination of variables, including genetics, age, number and specificity of islet autoantibodies, and metabolic measures, affect the risk of progression to clinical Stage 3 type 1 diabetes and account for the majority of type 1 diabetes risk(5,6).

Autoantibody screening for pre-clinical type 1 diabetes has been performed in natural history studies with a variety of designs(7). These designs include family studies, such as the Type 1 Diabetes TrialNet Pathway to Prevention Study (TrialNet)(8,9), birth cohort studies of genetically high-risk infants, e.g., The Environmental Determinants of Diabetes in the Young (TEDDY) study(9,10), and cross-sectional population screening of children, such as Fr1DA(11). Several methods to stratify individuals for future type 1 diabetes risk have been developed and tested in these studies. However, there are few assessments of whether models developed in one screening setting (e.g. longitudinal natural history studies) can be used in other approaches (e.g. cross sectional studies of relatives at risk). The transferability of a predictive model would support its validity, robustness, and generalizability, and facilitate accurate accurate risk prediction in a range of settings. Furthermore, the translation of predictive models into clinical research and practice requires freely accessible, user-friendly tools that provide individual risk prediction. Characteristics such as initial risk, age of screening, monitoring intensity and methods, genetic selection, and other study-specific variables that may further influence the accuracy, calibration, and general utility of prediction models. The availability of tools that incorporate multiple predictive variables for type 1 diabetes is currently limited but is common in other diseases (e.g., Q-risk)(12) and is likely to have utility as screening for type 1 diabetes expands.

We aimed to assess the generalisability of a type 1 diabetes prediction model developed in TEDDY, a birth cohort study that incorporates genetics, autoantibody status, age, and family history. We validated this model externally in the TrialNet study, a cohort of autoantibody positive relatives of individuals with type 1 diabetes. All TEDDY individuals were included in model development to ensure diverse risk profiles. In TrialNet, we focused on autoantibody-positive individuals to assess the generalisability of the model in this high-risk population, where autoantibody positivity is a key marker of progression to type 1 diabetes. Furthermore, we developed a type 1 diabetes risk prediction web application to quantify and visualize individual risk and allow use by at-risk individuals and their families, researchers, and clinicians.

## Methods

In summary, we developed a type 1 diabetes prediction model using data from the TEDDY study. For external validation, we used the TrialNet dataset. To adjust for the different selection criteria between the two studies, we used logistic recalibration. The steps taken are illustrated in Supplementary Figure 1.

### Participants

#### Discovery cohort: TEDDY

The TEDDY study followed since birth individuals from birth who carried a type 1 diabetes genetic risk based upon their HLA DR-DQ haplotype, as previously described(6). A total of 424,047 children were screened from the United States, Sweden, Germany, and Finland, with 8,676 children enrolled from September 2004 to February 2010. Of the 8,676 TEDDY enrollees, 7,798 were analysed on the basis of having available results for islet autoantibody testing and genotyping, as previously described(10,13). Risk at baseline can be defined from each landmark age.

#### External validation cohort: TrialNet Pathway to Prevention

TrialNet Pathway to Prevention is an observational study that prospectively follows autoantibody-positive relatives of people living with type 1 diabetes. Our analysis included 4,680 individuals enrolled between April 2004 and April 2023 who had at least one confirmed (positive on 2 consecutive tests) islet autoantibody and had a baseline oral glucose tolerance test (OGTT) within a year after testing autoantibody positive. Due to small number of adults (aged >18 years) with 3 autoantibodies these people were grouped with 2 autoantibody positive individuals into a >2 autoantibody group for calculation of individual risk prediction analyses. Risk at baseline for TrialNet individuals is defined as the risk from their first recorded visit.

All study participants gave informed consent, and the studies were approved by the ethics committee at each site.

### Procedures

#### Study Protocol:

TEDDY participants were monitored every 3 months until 4 years of age with autoantibody tests, and oral glucose tolerance tests (OGTTs) in autoantibody positive individuals, and then either 3 or 6-month intervals depending on autoantibody status. After age 4, autoantibody-negative children were assessed every 6 months while children with any positive autoantibody remained on quartely visits until 15 years of age or until the onset of clinical type 1 diabetes(13,14).

TrialNet participants were monitored at 6 or 12-month intervals (with autoantibody and OGTT testing) depending on their estimated risk. Single autoantibody-positive individuals were followed on an annual basis. Multiple autoantibody-positive were usually followed on a semi-annual basis(15). All individuals were monitored until progression to type 1 diabetes or entered into a prevention clinical trial.

#### Autoantibody assays:

TEDDY and TrialNet participants were tested for autoantibodies to glutamic acid decarboxylase (GADA), insulin (micro-insulin antibody assay [mIAA]), and insulinoma associated antigen 2 (IA-2A). GADA, mIAA, and IA-2A autoantibodies were used in the original model development, and therefore to accurately assess generalisability across datasets, we assessed the positivity of these autoantibodies. GADA, mIAA, and IA-2A were measured by radioimmunoassay in the TrialNet Core Laboratory at the Barbara Davis Center for TrialNet individuals. Autoantibodies for TEDDY participants were measured in two separate core laboratories, as previously described(16).

#### Diagnosis of Type 1 Diabetes:

Type 1 diabetes was diagnosed based on hyperglycemia, including fasting plasma glucose ≥7.0 mmol/L (≥126 mg/dL), 2-hour plasma glucose ≥11.1 mmol/L (≥200 mg/dL) after 75 g oral glucose, or HbA1c ≥6.5% (≥48 mmol/mol), with diagnoses confirmed if symptomatic type 1 diabetes or testing confirmed on two separate occasions if asymptomatic.

#### SNP Genotyping and Genetic Risk Score (GRS) computation:

TEDDY participants were genotyped on an Illumina Infinium ImmunoChip SNP array. Prior to imputation, quality control filtering was conducted on SNP variants based on missing genotype rates (<1%), Hardy-Weinberg equilibrium (p<1×10^−6^), and minor allele frequency (<1%). The ImmunoChip data was imputed using the 1000 Genomes reference panel. Sharp et al. 2(17) includes 42 SNPs which were directly genotyped and we imputed 25 SNPs, 6 non-HLA and 19 HLA. 21 SNPs had an r^2^ ≥ 0.75 and four SNPs were imputed with r^2^ = 0.358 – 0.544.

TrialNet participants were genotyped on an Illumina Infinium T1DExomeChip SNP array. TrialNet participants genotyping data was imputed using the TOPMed reference panel as previously described(18). Sharp et al. GRS(17) includes 30 SNPs which were directly genotyped and we imputed the remaining 37 SNPs, 21 non-HLA and 5 HLA, using the TOPMed reference panel(19,20) and Michigan Imputation Server(21), respectively. The median r^2^ of the non-HLA SNPs was 0.997 (min = 0.858, max = 0.999) and the median r^2^ of the HLA SNPs was 0.998 (min = 0.925, max = 0.999). Code to generate the HLA interaction part of the type 1 diabetes GRS is freely available online (https://github.com/sethsh7/hla-prs-toolkit).

### Statistical Analysis

#### Model Training

We aimed to refine a previously published prediction model(6), utilising the variables and methodology. The original prediction model combined the GRS(17), number of positive autoantibodies, and the presence of a first-degree relative (FDR) with type 1 diabetes. Using a stratified Cox proportional hazards model with a landmarking approach, the new stratified prediction model combines these variables and allows scores to be updated as new information becomes available(6,22). In the proposed stratified prediction model, individual-specific variables proportionally change the mean risk of type 1 diabetes, with the mean risk representing the average risk of the entire cohort without considering these individual variables.

Predictors of disease progression can change over time, which can violate the proportional hazards (PH) assumption in a Cox proportional hazards model. Prior to seroconversion, genetics and family history are the most predictive variables of type 1 diabetes and, therefore, have the most influence on diabetes risk(5). However, when an individual has stage 1 or 2 type 1 diabetes, metabolic and autoantibody features are most predictive variables(5). The shift in the weight of predictive variables depending on stage of type 1 diabetes violates the PH assumption, potentially producing misleading results(23). To address this, we incorporated stratification by the number of autoantibodies to account for heterogeneity between predictors by stage and ensure more accurate modelling of the disease progression(24,25).

#### External Validation

We used the log-rank test to assess the difference in disease-free survival probabilities between studies, TEDDY and TrialNet. Due to the large differences within the study design, TrialNet (the validation dataset) included individuals over a larger age range than TEDDY (the discovery dataset). We divided TrialNet participants into three categories characterised by their age at screening and date of autoantibody positivity. The first category included individuals most similar to TEDDY, i.e., aged 7 years and younger (n=1,310). The second category included TrialNet children screened between (and including) 8 and 17 years of age (n=1,794). The third category included all TrialNet autoantibody positive adults (i.e., 18 years of age and older) (n=963). We used a landmark approach as previously described(6) (see Supplementary Method for details).

### Evaluation of Predictive Performance

We measured two aspects of predictive performance: discrimination and calibration. Discrimination, as measured by the area under the time-dependent receiver operating characteristic curve (ROC-AUC)(26), measures the extent to which the model can predict an event at future horizons. We computed ROC-AUC using three-fold cross-validation, repeated ten-fold to reduce overestimation of model performance. ROC-AUC was evaluated at 1, 3, and 5 years in both cohorts and due to the extended follow-up in TEDDY, ROC-AUC was additionally evaluated at 8 years in TEDDY. Calibration measures the agreement between the predicted risk and the observed outcomes. We use smoothed calibration curves(27) and survival Brier score(28) to assess model calibration. We calculated the Brier score also using three-fold cross-validation, repeated ten-fold, the same internal validation methodology as ROC-AUC. We assessed the predictive performance difference in the pre-defined TrialNet age groups, with paired t-tests to determine the signficance between models.

### Recalibration for Cohort Differences

As expected, differences in risk at baseline are apparent between the discovery (TEDDY) and validation (TrialNet) datasets, consistent across the landmark ages in TEDDY. These two studies have differing eligibility criteria, and proportions of individuals progressing to disease. For example, since we only selected autoantibdy positive TrialNet participants, they are at higher risk of progressing to clinical type 1 diabetes than TEDDY participants. This difference causes the model developed in TEDDY to underestimate the risks in TrialNet participants. Therefore, to adjust for differences in both the baseline risk and rate of progression between the discovery (TEDDY) and external validation (TrialNet) cohort, we performed logistic recalibration to the stratified Cox model. Logistic recalibration fits the linear predictors from the original model as the only covariate per strata in a Cox proportional hazards model(29), adjusting baseline risk and coefficients by a common factor. Results were computed with R version 4.3.1 (2023-06-16)(30) with the packages survival(31) and timeROC(26). P-values<0.05 were considered statistically significant.

### Availability of data and materials:

The code used in the current study to analyze the datasets is available online (https://github.com/TemplemanErin/recalibration).

## Results

### Participant baseline characteristics and progression to stage 3 type 1 diabetes

The analysis included 7,798 TEDDY participants with a median follow-up period of 9.3 years, and 4,086 TrialNet participants (mean ± SD age 14.9 ± 12.2 years; 32% ≤ 7 years of age) with a median follow-up period of 4.8 years. Type 1 diabetes developed in 305 TEDDY participants (4%) and 1,373 TrialNet participants (34%) during the follow-up. Further characteristics of the TEDDY and TrialNet participants, categorised by age-group, are detailed in Table 1.

Survival analysis demonstrated differences in absolute risk of progression between the two cohorts. As expected, TrialNet participants demonstrated a higher risk of progression to type 1 diabetes risk than TEDDY participants (5-year type 1 diabetes risk; TrialNet 35% [95% CI 33,37]; TEDDY: 2% [95% CI 2,3]; p<0.001).

Significant differences persisted when the analysis was conducted with stratification by autoantibody status compared to individuals from TEDDY. Among single autoantibody-positive individuals, those in TrialNet had around a 10% higher risk of progressing to stage 3 clinical disease than those in TEDDY (TrialNet: 19% [17,21]]; TEDDY: 12% [7,17]; p=0.08). The risk of progression of individuals present with two autoantibodies is also higher in TrialNet, by around 15% (TrialNet: 48% [44, 51]; TEDDY: 33% [23,42]; p=0.04). However, we observed no difference in probability of the progression between the two studies for participants with three positive autoantibodies (TrialNet: 61% [56,65]; TEDDY: 67% [56,75]; p=0.40) (Supplementary Figure 3 and Supplementary Table 2).

The GRS was a stronger discriminator as an individual variable in TEDDY when compared to TrialNet (ROC-AUC TEDDY: 0.73 [95% CI 0.71,0.76]; TrialNet: 0.64 [95% CI 0.62,0.67]; p < 0.001). However, when comparing individuals with similar characteristics (e.g., autoantibody positive), discrimination was similar across the two cohorts (ROC-AUC TEDDY: 0.64 [0.58,0.71]; TrialNet: 0.64 [0.62,0.67]; p>0.5).

### Validation of stratified prediction model in autoantibody positive TrialNet individuals

We stratified the TEDDY model by autoantibody status to address the imbalance of autoantibody-negative to autoantibody-positive individuals between cohorts. We first assessed the stratified model’s performance and calibration in the TEDDY cohort. In this study, discrimination measures how well a model can distinguish between participants who will develop T1D from those who will not, while calibration assesses how closely the predicted probabilities match T1D onset. The model performed well (ROC-AUC: 0.75–0.98 for all time horizons (Figure 3A)). We then assessed discrimination in TrialNet and found the model was similarly discriminative when comparing TrialNet individuals with individuals with similar characteristics from TEDDY (e.g., autoantibody positive) (mean 3-year ROC-AUC TrialNet: 0.712, TEDDY: 0.756, p= 0.21; Supplementary Figure 4B and Supplementary Figure 4C). As expected, explained by different criteria selection, we observed consistent underestimation of the risk of type 1 diabetes in TrialNet (Supplementary Figure 5). With an observed probability of 50%, predicted risk estimates are underestimated by 5-fold (aged under 8 = 10%, and aged between 8 and 17 = 9%), and 2.5-fold in adults (aged over 18 = 20%).

### Recalibration of prediction model in autoantibody positive TrialNet individuals

**Figure 1:**
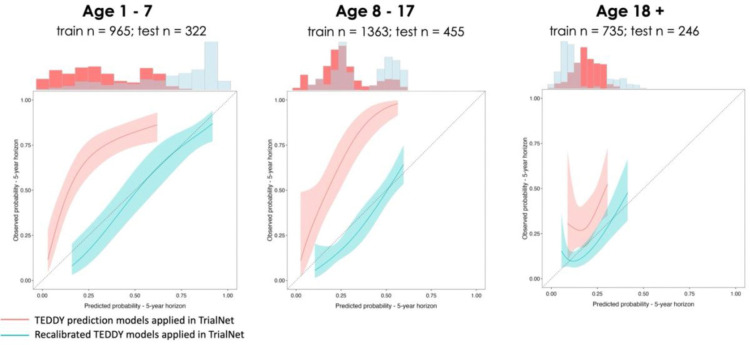
Calibration of stratified TEDDY prediction models in TrialNet if applied directly and recalibrated to external, validation dataset. Legend: Recalibration of stratified TEDDY prediction models in TrialNet improves the calibration of prediction estimates and maintains predictive ability. Larger confidence intervals are typically due to fewer individuals in the test cohort compared to the training cohort, being predicted at these probabilities, therefore increasing the uncertainty around the estimate. The dotted line represents perfect calibration, where predicted probabilities align with the observed outcomes.

Next, we asked the question of whether a simple recalibration could take advantage of cohort similarities and modify the model for use across studies such as TEDDY and TrialNet. We observed that logisitic recalibration significantly improved the model’s performance, removing the underestimation of risk and resulting in more accurate and calibrated predictions (Fig. 1).

**Figure 2:**
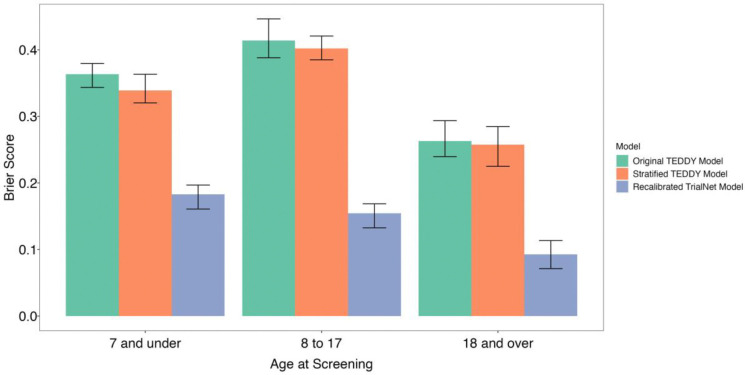
Accounting for differences in datasets through recalibration allows for a lower Brier score, i.e., a higher degree of calibration of estimates. Legend. Bar chart with 95% confidence intervals represented as error bars of the estimate of the Brier score of the TEDDY-derived linear and stratified prediction models applied in TrialNet and the internally-validated recalibrated model, accounting for the differences between datasets. For three-fold validation, each age group is split into three, and therefore the brier score is tested in a third of each age group (Age 7 and under: 1,287 participants; Age 8 to 17: 1,818 participants; Age 18 and over: 981 participants).

We observed significantly reduced Brier score values indiciating improved calibration in the recalibrated model compared to either the stratified or original (linear) TEDDY-derived models (Figure 2).

The difference between the Brier Score in the recalibrated model compared to the stratified model was largest in individuals aged between 8 and 17 years old (stratified Brier Score = 0.403 [95% CI 0.375,0.430]; recalibrated Brier Score = 0.161 [95% CI 0.144,0.166]; difference = 0.2422). A lower Brier score indicates better model accuracy, where here there is an 85% decrease representing signficant prediction reliability. Discrimination of the recalibrated model across age groups was not significantly different (p=0.29) (Supplementary Figure 6).

### Individualised Risk Prediction: Development of a risk calculator tool

We generated individualised type 1 diabetes risk estimates using the predictors of the recalibrated prediction model (number of autoantibodies, GRS, and presence of an affected first-degree relative) and the age group of the individual (Supplementary Table 3). We observed a strong impact of GRS on disease prediction, even in TrialNet, where all individuals are positive for autoantibodies and have a positive family history of type 1 diabetes. For a 7-year-old child with a single autoantibody, having a 5^th^ percentile GRS predicts an 18% 5-year risk of developing type 1 diabetes, while a 95^th^ percentile GRS would substantially raise the 5-year risk to 56%.

**Figure 3:**
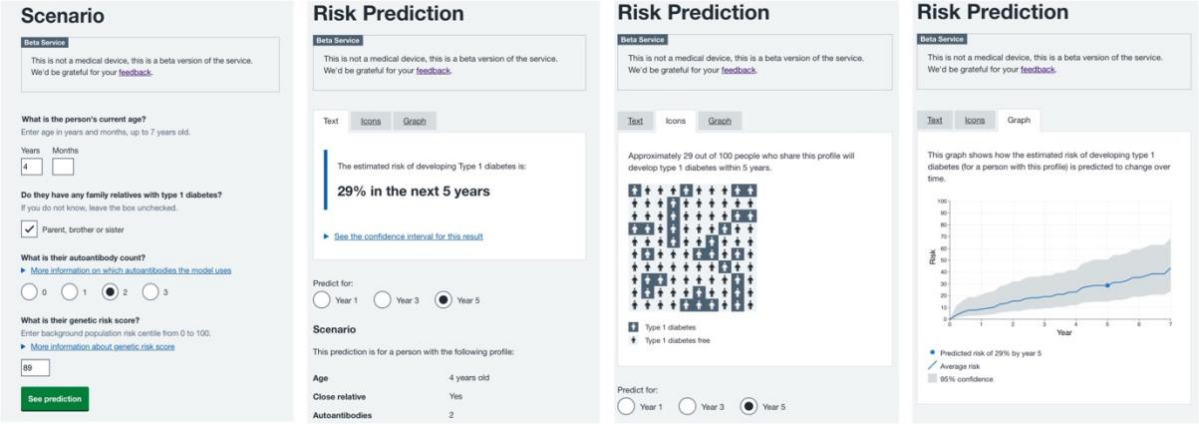
Risk calculator and example results. Legend: An example output from the stage 3 type 1 diabetes risk calculator displaying the scenario inputs required, and the three visualisations available.

To facilitate individualised risk prediction, we developed an application where an individual’s features can be entered to generate their type 1 diabetes risk estimate using the recalibrated model (https://t1dpredictor.diabetesgenes.org(32)). Type 1 diabetes risk is displayed through a variety of visualisations, estimated risk with a confidence interval, icon array plot, and a line graph displaying the increasing risk over time (Figure 3).

## Discussion

We tested a type 1 diabetes prediction model developed in a birth cohort and found the model was discriminative in a cohort of cross-sectionally ascertained, autoantibody-positive relatives of individuals with type 1 diabetes. As expected, the model required recalibration. We observed differences by age that were accounted for by a landmark approach. However, predictions in adults were less well calibrated than in children under 18. We developed a type 1 diabetes prediction web application, that allows application of the TEDDY and recalibrated TrialNet models to individuals currently being monitored in screening studies. Translation of validated models into routine care will require easy-to-use interfaces such as this, linked to electronic health record biomarkers and clinical data.

Our study builds on previous work that showed accurate discrimination and internal validation of a combined risk model in TEDDY, one of the largest and most comprehensive natural history studies of infants followed from birth. We stratified the TEDDY model by autoantibody status to address the imbalance between the larger number of autoantibody negative individuals and the smaller number of autoantibody positive individuals in the discovery dataset (TEDDY), compared with the validation dataset (TrialNet), where inclusion required the presence of one or more autoantibodies in this cohort. This was important because a model mainly trained on autoantibody negative individuals would likely not work well in autoantibody positive individuals, and predictive variables have varying impact across diabetes stages. Our stratified model had higher weights for family history and GRS in single autoantibody positive individuals compared to multiple autoantibody positive individuals, highlighting that genetic risk has a greater influence before the onset of stage 1 type 1 diabetes, which is consistent with our previous findings in TrialNet(5). We used the same variables for all models, with different coefficients dependent on autoantibody status; however, others have shown the importance of metabolic variables once individuals have stage 1 type 1 diabetes(33).

The good discrimination of the original TEDDY model in the TrialNet dataset highlights that, in keeping with existing knowledge, the main and strongest predictors of type 1 diabetes are consistent across many study designs(34–36). We note, as previously described(6), that the level of discrimination is less when compared to screening or classification settings where there has been no genetic pre-selection to rule out large numbers of low risk individuals. However, the similar discrimination between TrialNet individuals and TEDDY individuals with similar characteristics highlights that the features of the model have validity despite the different age ranges and selection of the discovery and validation cohorts.

We recalibrated the TEDDY model for the TrialNet study to enhance its utility across various screening settings. Given the differences in baseline risk, logistic recalibration allowed us to significantly improve calibration by using a straightforward adjustment. While developing a new model in one screening setting might achieve similar results, it requires substantially more data and time. In contrast, recalibration leverages existing work, yielding accurate predictions with a more robust and transferable model that can be effectively applied. As screening for type 1 diabetes becomes more widespread, recalibration of existing models offers a practical and timely solution for ensuring accurate predictions in the wave of screening studies now being initiated worldwide.

Age of assessment is critical to the prediction of type 1 diabetes; we accounted for age in the original, TEDDY-derived model, by taking a landmark approach to modelling(6), as others have done(37). The age landmark approach allows for differences in risk of progression depending on age, with a higher likelihood of rapid progression in younger children(38). Good discrimination and accurate calibration were achieved once differences in baseline risk had been accounted for; our results imply accurate prediction using the recalibrated model in screening studies for children up to age 18. We had less data over the age of 18 and found model estimates to be discriminative but calibrated slightly less well. We have limited data for this age group, and further studies are needed to fully understand adult-onset type 1 diabetes and the variables that can help refine predictive models.

Our model collapsed information from autoantibodies into a categorical variable of autoantibody number (0,1, 2, or 3), due to the size of the discovery dataset and the relative importance of autoantibody number in risk prediction and staging. Other features, such as autoantibody type and titre, may be important for risk prediction and warrant further investigation. An important consideration is the ease of assessment; for example, autoantibody testing assays across the world are compared by the Islet Autoantibody Standardisation Programme (IASP)(39). Assay performance against a standard set of positive and negative samples is compared across commercial and academic assays. However, titre is not harmonised, and not all studies measure the same autoantibodies. Therefore, this is a trade-off of precision of assessment against the generalizability and practicality of testing in the public health setting.

The generation of the application represents an easy-to-use tool to help understand an individual’s risk and assist in decision making. This creates an interface for clinical researchers, clinicians, families, or individuals themselves to generate type 1 diabetes risk estimates. This may be helpful for communication of risk, and for decisions on the frequency of individual follow up. This approach of generating an app or calculator has been successful in many other settings, maturity-onset diabetes in the young (MODY) and cardiovascular disease, which are both used to make clinical decisions(40,41).

### Limitations

Recalibration of the TEDDY-derived prediction model improved the calibration of estimates and resulted in well calibrated estimates in all age groups. Discrimination could be improved by additional predictors and their interaction(42), such as the inclusion of metabolic variables particularly for the later stages.

Most of the individuals in both cohorts are of European ancestry; in the future, it is important to address this imbalance by including individuals from diverse ancestries and geographies to ensure prediction models are valid for all. We used T1D GRS percentiles derived from a UK population for individualized risk predictions, assuming that the GRS distributions are similar across the UK and US. However, it has been previously demonstrated that GRS distributions can vary across populations(43). Validating these differences and adjusting percentiles accordingly are necessary to ensure accurate and equitable predictions.

Recalibration is common within disease prediction, making adjustments to improve prediction accuracy and reliability as patient populations evolve which have been utilised in many other disease settings including colon cancer and COVID-19(44,45). Besides logistic recalibration, other methodologies include temporal recalibration, which adjusts prediction over time, bayesian updating which updates the parameters with new data while retaining prior information, and dynamic updating that continuously updates predictions as new data becomes available. However, these methodologies were not applied in this study as we aimed to implement a simpler update to the model due to available data constraints.

The recalibration of the prediction models uses baseline variables, as described in the methods. This allows for simplification of type 1 diabetes risk calculation, making risk accessible when screening individuals at a single timepoint when no historical data is available. Additional risk information, from longitudinal follow-up, can be considered to further improve discrimination and calibration of prediction models, such as joint modelling or models utilising historical data, like hemoglobin A1c(46,47). However, these approaches require data to be obtained from multiple visits and therefore require more information and potentially more computing resource(48).

## Summary

We externally validated a type 1 diabetes prediction model with recalibration of risk to account for a different screening scenario. Our study highlights that models of disease risk are likely to be broadly generalisable with modest recalibration required depending on the screening setting. Importantly, we developed an interactive tool that allows clinicians and screened individuals and/or families to visualise risk, and this may be important for accurate communication of future type 1 diabetes risk in screening studies.

## Figures and Tables

**Table 1.** Table of summary characteristics of TEDDY individuals and TrialNet individuals, split by age-group.

		TEDDY n = 7,798	TrialNet, split by age at screening (yrs)		
			Age 1 – 7 n = 1,287	Age 8 – 17 n = 1,818	Age 18+ n = 981
**Cohort selection**		High genetic risk individuals followed	Cross-sectional screening of autoantibody-positive relatives of individuals with type 1 diabetes		
**Developed type 1 diabetes**		305 (3.9%)	600 (46.6%)	601 (33.1%)	172 (17.5%)
**Sex (% female)**		3,832 (49.1%)	598 (46.5%)	811 (44.6%)	629 (64.1%)
**First-degree relative with type 1 diabetes**		886 (11.4%)	1,101 (85.5%)	1,587 (87.3%)	950 (96.8%)
**Progression to multiple autoantibodies**		452 (5.8%)	989 (76.8%)	1,207 (66.4%)	369 (37.6%)
**GRS [Mean (SD)]**		14.3 (1.30)	13.5 (2.09)	13.2 (2.10)	12.7 (2.21)
**Follow-up [median years (IQR)]**		9.5 (5.2; 11.1)	5.8 (3.0; 9.3)	4.5 (2.0; 7.6)	4.4 (1.9; 8.0)
**Race**	**Hispanic, regardless of race**	571 (7.3%)	102 (7.9%)	203 (11.2%)	94 (9.6%)
	**White, non-Hispanic**	4,671 (59.9%)	1,043 (81.0%)	1,413 (77.7%)	790 (80.5%)
	**African American, non-Hispanic**	67 (0.9%)	37 (2.9%)	45 (2.5%)	23 (2.3%)
	**All other races, non-Hispanic**	151 (1.9%)	21 (1.6%)	25 (1.4%)	16 (1.6%)
	**Unknown** *	2,338 (30.0%)	84 (6.5%)	132 (7.3%)	58 (5.9%)

Values are presented as counts numbers (percentage of group) unless otherwise stated. Type 1 diabetes is abbreviated to “T1D” and genetic risk score is abbreviated to “GRS”.

* indicates missing Race or Ethnicity data; the percentage is higher in TEDDY due to a differential collection and reporting within Europe.

## Data Availability

The code used in the current study to analyze the datasets is available from the corresponding author upon reasonable request.

## List of Abbreviations

CI: Confidence Interval
DASP: Diabetes Autoantibody Standardisation Programme
et al.: and others
FDR: First-Degree Relative
g: grams
GAD: Glutamic Acid Decarboxylase
GRS: Genetic Risk Score
HLA: Human Leukocyte Antigens
IA-2A: Insulinoma Associated Antigen 2
IQR: Interquartile Range
Max: maximum
mg/dL: milligrams per deciliter
mIAA: Micro-Insulin Antibody Assay
min: minimum
mmol/L: millimoles per lite
mmol/mol: millimoles per mole
MODY: Maturity onset diabetes of the young
OGTT: Oral Glucose Tolerance Test
PH: Proportional Hazards
ROC: AUC Area under Receiver Operating Characteristic Curve
SD: Standard Deviation
SNP: Single Nucleotide Polymorphism
TEDDY: The Environmental Determinants of Diabetes in the Young
U.S.: FDA Food and Drug Administration
